# Sialoblastoma of the Minor Salivary Glands: A Case Report

**DOI:** 10.1155/crid/5435950

**Published:** 2026-06-20

**Authors:** Giorgio Novelli, Giulia Mangini, Chiara Picozzi, Camillo Di Bella, Gabriele Canzi, Davide Sozzi

**Affiliations:** ^1^ Maxillofacial Surgery Unit, Fondazione IRCCS San Gerardo dei Tintori, Monza, Italy; ^2^ School of Medicine and Surgery, University of Milano-Bicocca, Milan, Italy, unimib.it; ^3^ Postgraduate School of Maxillofacial Surgery, University of Milano-Bicocca, Milan, Italy, unimib.it; ^4^ Department of Pathology, Fondazione IRCCS San Gerardo dei Tintori, Monza, Italy; ^5^ Maxillofacial Surgery Unit, Department of Neuroscience–Head and Neck, ASST GOM Niguarda, Milan, Italy

**Keywords:** case report, minor salivary glands, pediatric tumors, salivary gland neoplasms, sialoblastoma

## Abstract

**Background:**

Sialoblastoma is a rare malignant epithelial tumor of the salivary glands, predominantly affecting children, with approximately 60 cases reported in the literature. It most commonly involves the parotid gland but may also arise from the submandibular and minor salivary glands. Clinically, it usually presents as a painless mass, with metastatic potential being uncommon.

**Case Presentation:**

We report the case of a 9‐year‐old girl, otherwise in good general health, presenting with a painless solid mass in the buccal mucosa. Diagnostic evaluation included fine‐needle aspiration, incisional biopsy, and magnetic resonance imaging (MRI) imaging. The lesion was surgically excised, and the resulting defect was reconstructed using a Bichat’s fat pad flap. Histopathological examination confirmed sialoblastoma originating from the minor salivary glands.

**Outcomes:**

Complete surgical excision with clear margins was achieved. Postoperative follow‐up with MRI and chest X‐rays demonstrated no evidence of recurrence or metastasis. No adjuvant therapy was required given the complete resection and favorable histological features.

**Discussion:**

Pediatric sialoblastoma is extremely rare, and evidence regarding management is limited. Complete surgical resection with clear margins remains the gold standard. Although recurrence occurs in approximately 25% of cases, early diagnosis and intervention are associated with excellent prognosis, with a reported 5‐year survival rate of 95.5%. Chemotherapy or radiotherapy is reserved for advanced or recurrent cases due to potential long‐term adverse effects.

**Conclusion:**

The rarity of sialoblastoma limits the development of standardized diagnostic and therapeutic protocols. Treatment should be individualized based on patient and tumor characteristics; however, surgical excision remains the cornerstone of management.

## 1. Introduction

Sialoblastoma is a rare primitive epithelial tumor of the salivary glands, with only about 60 cases reported in the literature to date [1, 2]. First described in 1966 by Vawter and Teff [3], the term “sialoblastoma” was introduced by Taylor[4] in 1988 to highlight its salivary origin and dysontogenic nature [5], it was reclassified by the WHO in 2005 as a malignant epithelial neoplasm, despite its uncertain malignant potential [6]. Sialoblastoma is typically diagnosed in infancy, with 80% of cases being congenital, although it can present up to the second decade of life. The parotid gland is most commonly affected (45.2%). Clinically, it often appears as a palpable, sometimes painful lesion, with rare severe neurological symptoms [2]. Metastases are rare, occurring exclusively in parotid cases and involving the lungs or laterocervical lymph nodes [7].

## 2. Case Presentation

We report the case of a 9‐year‐old girl, otherwise in good general health, who was referred to our care center by her dentist for a right genial, roundish mass near the masseter muscle. The patient had no relevant past medical or family history. The lesion was first noticed in the summer of 2023, and our initial evaluation took place in October of the same year. The mass was painless, solid on palpation, measured approximately 15Å~ 10 mm at its greatest diameter, was nonpulsatile, and mildly tender. The genienal mucosa and skin appeared intact. Initial cytological examination via fine needle aspiration, performed during our first evaluation, yielded a nondiagnostic result. An incisional biopsy was carried out in November 2023, and subsequent histopathological examination revealed a myoepithelial‐differentiated neoplasm likely originating from the minor salivary glands, demonstrating an infiltrative growth pattern towards the stroma. Microscopic analysis depicted tissue fragments partially occupied by minor salivary glands, showcasing proliferation of ductal/cord‐like structures lined by small, monomorphic epithelial cells within a fibroadipose stroma. Additionally, larger polygonal and spindle‐shaped cellular elements with abundant pale cytoplasm and round or oval nuclei were observed, organized in paucicellular aggregates with infiltrative behavior towards the surrounding stroma. Notably, no unequivocal features of perineural invasion were identified. Immunohistochemical staining revealed intense reactivity for S100 protein, weak staining for cytokeratin pool, smooth muscle actin, and calponin, while staining for EMA and p63 was negative.

**Figure 1:**
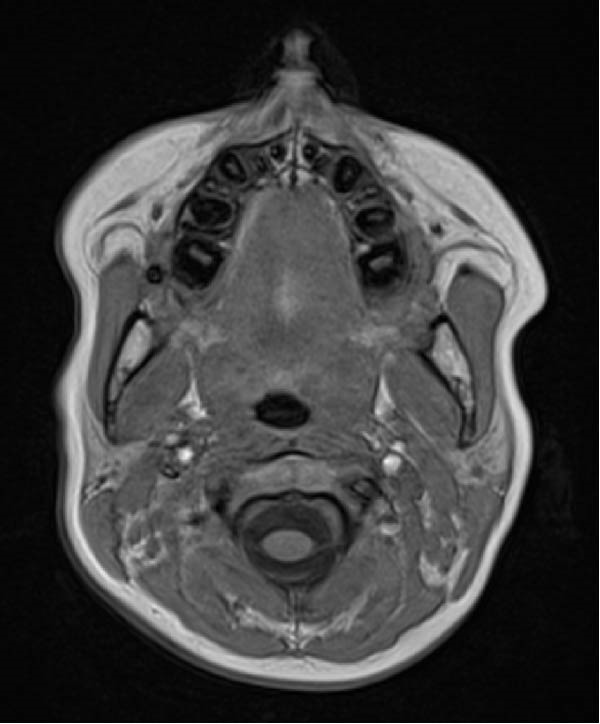
T1‐weighted MRI image.

Following this, the patient underwent a comprehensive diagnostic evaluation with magnetic resonance imaging (MRI) imaging. No MRI scan of the untreated primary lesion was available. The examination revealed a focal round‐shaped signal alteration of approximately 3 mm within the right cheek located between the most posterior dental element of the upper arch and the horizontal branch of the hemimandible. The finding was consistent with the residual lesion at the previous biopsy site, without diffusion restriction or contrast enhancement. Notably, the submandibular and parotid glands exhibited a normal appearance, with no pathological lymph node swellings observed within the explored volume (Figure 1). Surgical excision of the neoformation was performed in December 2023, 2 weeks after the results of MRI and incisional biopsy, under general anesthesia via an endoral approach, followed by reconstruction of the residual deficit at the genial level using a buccal fat pad (Figure 2a–d).

Figure 2(a) Right buccal mass ⟶ clinical aspect, (b) preoperative planning, (c) harvesting the buccal fat, (d) postoperative result.

**Figure:**
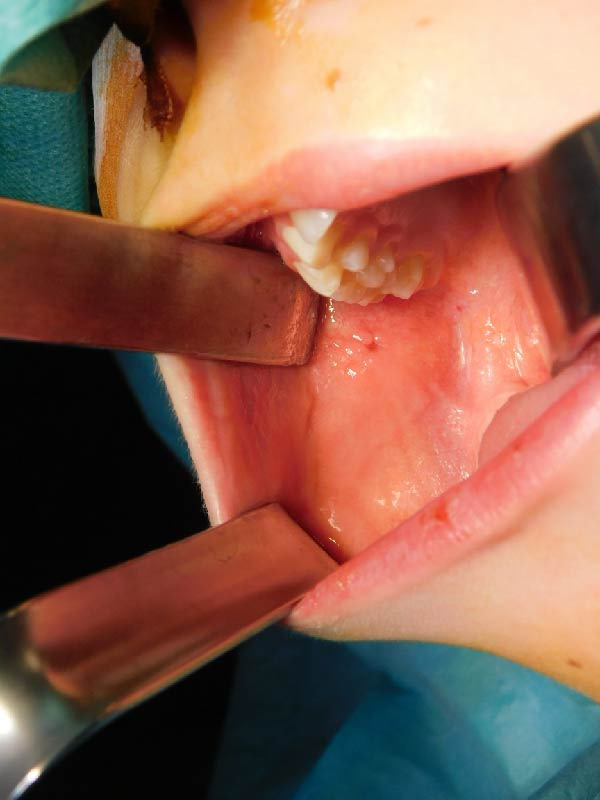
(a)

**Figure:**
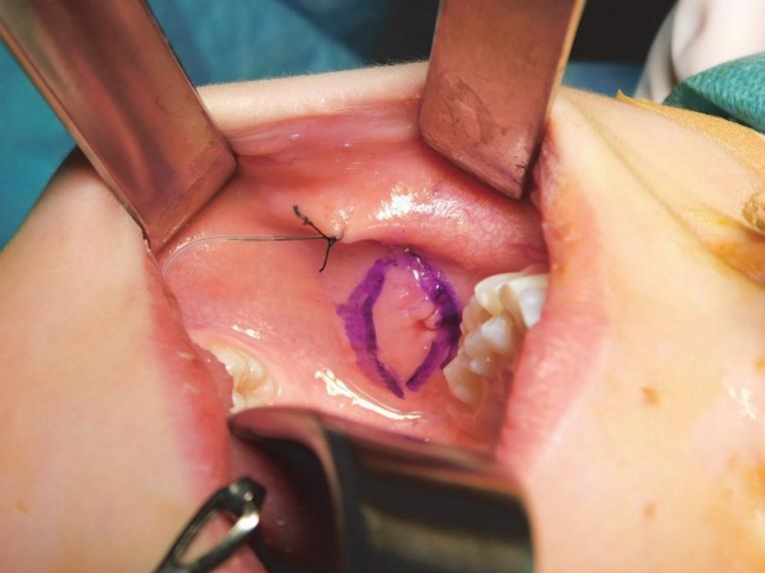
(b)

**Figure:**
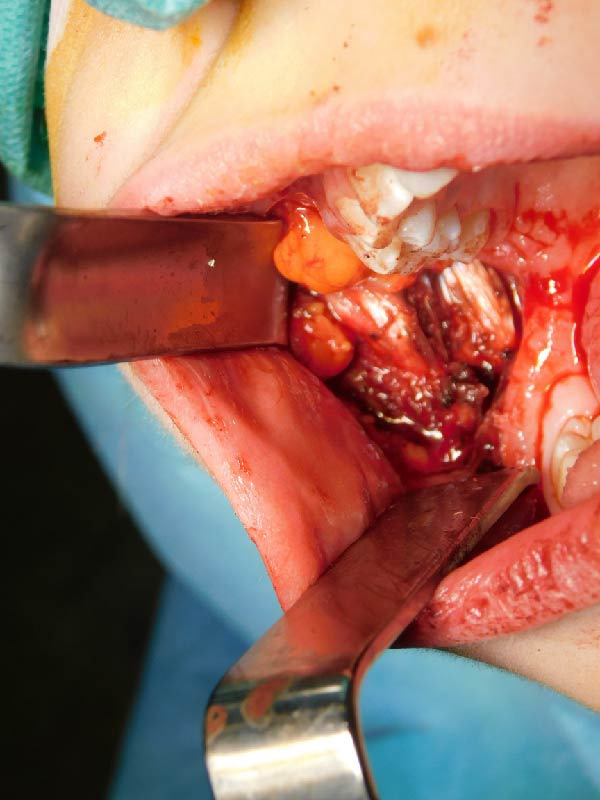
(c)

**Figure:**
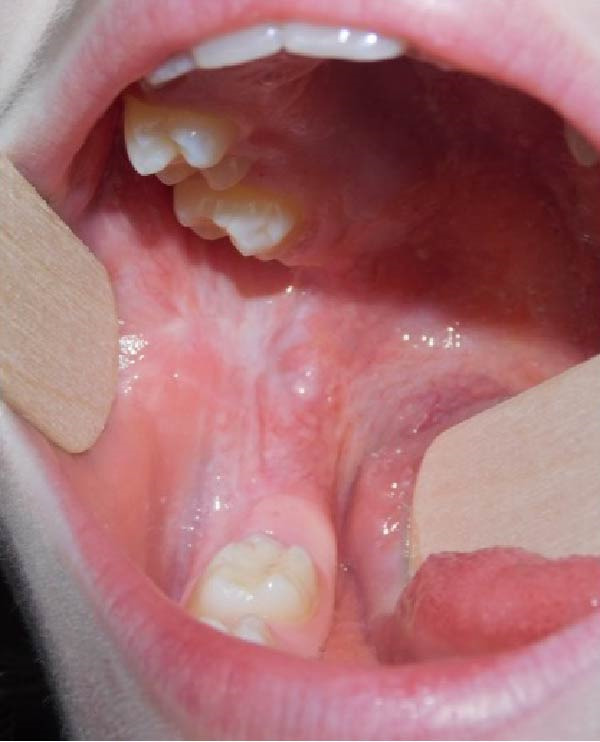
(d)

The excised tissue was subjected to definitive histological examination. The specimen was also sent for further analysis to the National Cancer Institute. The final report revealed mucosal and soft tissue of the cheek with aggregates of salivary glands and adjacent proliferation of ductal/cord‐like structures predominantly comprising small monomorphic epithelial elements. The neoplastic population displayed mild cytological atypia, absence of necrosis, irrelevant mitotic index, and a low proliferation rate. Immunophenotypic analysis indicated positivity for CKAE1/AE3, CK7 (focal), and S100, while the basal/myoepithelial component was positive for P40, P63, and smooth muscle actin (focal). The neoplasm in the excision specimen shows an infiltrative growth pattern into the surrounding stroma, with perineural involvement. The proliferation index (Ki67) was low, consistent with a diagnosis of sialoblastoma, a basaloid neoplasm of salivary glands (Figure 3a–d,f–h).

Figure 3(a, b) Proliferation of ductal/cord structures of epithelial elements with mild atypia and absence of necrosis (H and E staining, magnification 20x); (c) focal involvement of small nervous structures by the neoplasm (H and E staining, magnification 40x); (d) perineural invasion (H and E staining, magnification 20x); (e) proliferation of ductal/cord structures of epithelial elements with mild atypia (H and E staining, magnification 40x); (f) S100 protein (magnification 40x); (g) pan‐cytokeratin (magnification 40×); and (h) Ki‐67 (magnification 20x).

**Figure:**
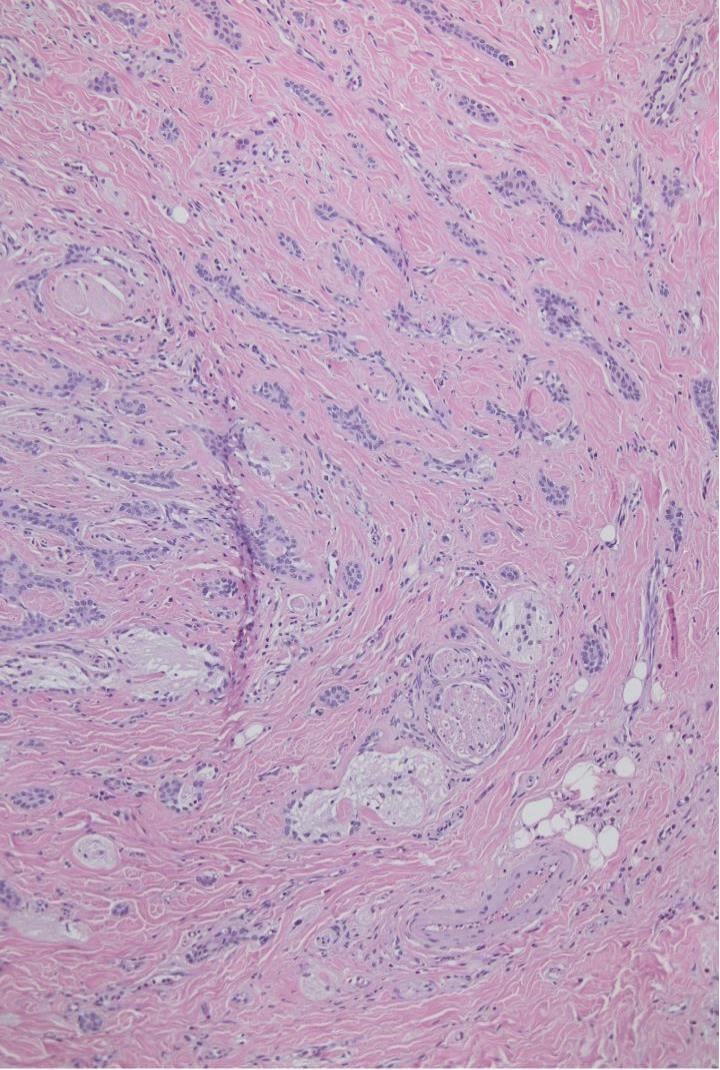
(a)

**Figure:**
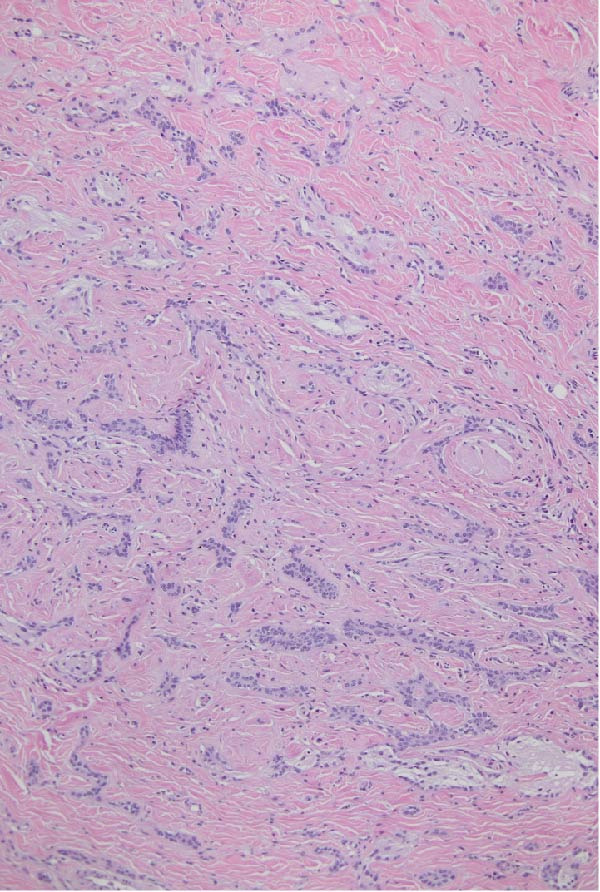
(b)

**Figure:**
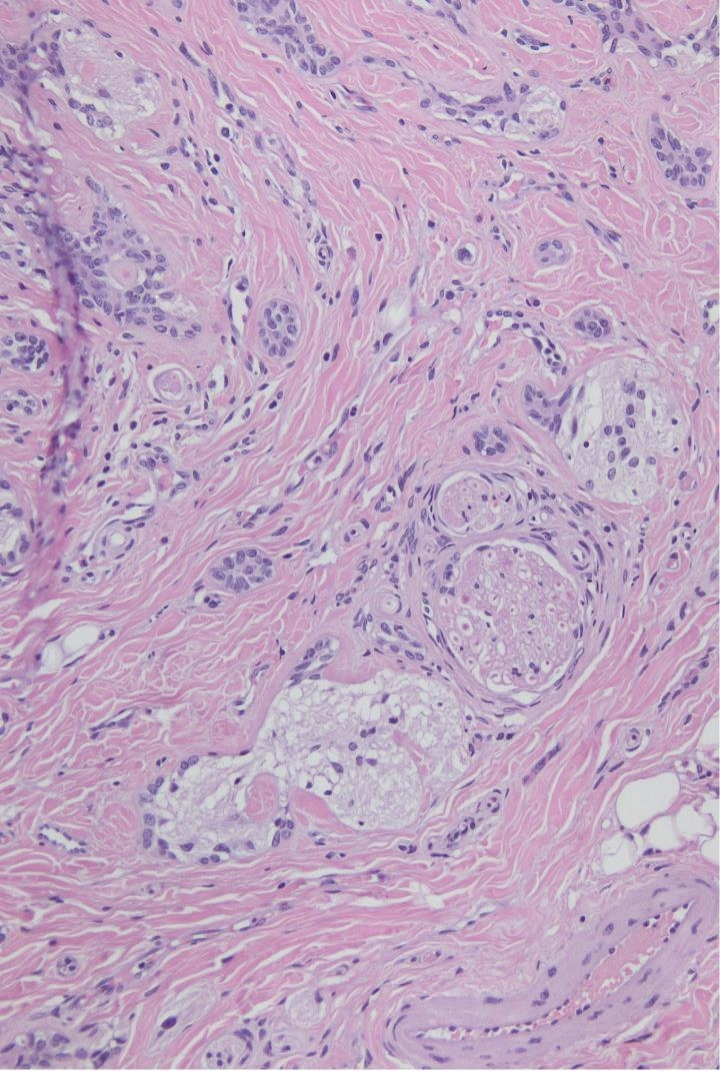
(c)

**Figure:**
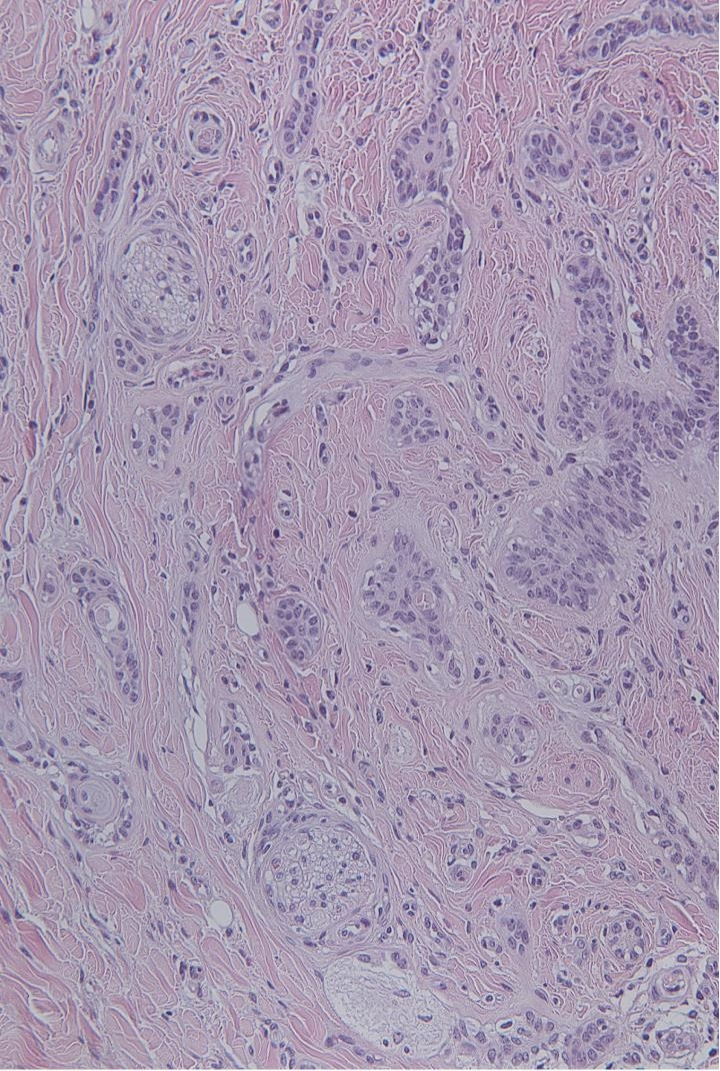
(d)

**Figure:**
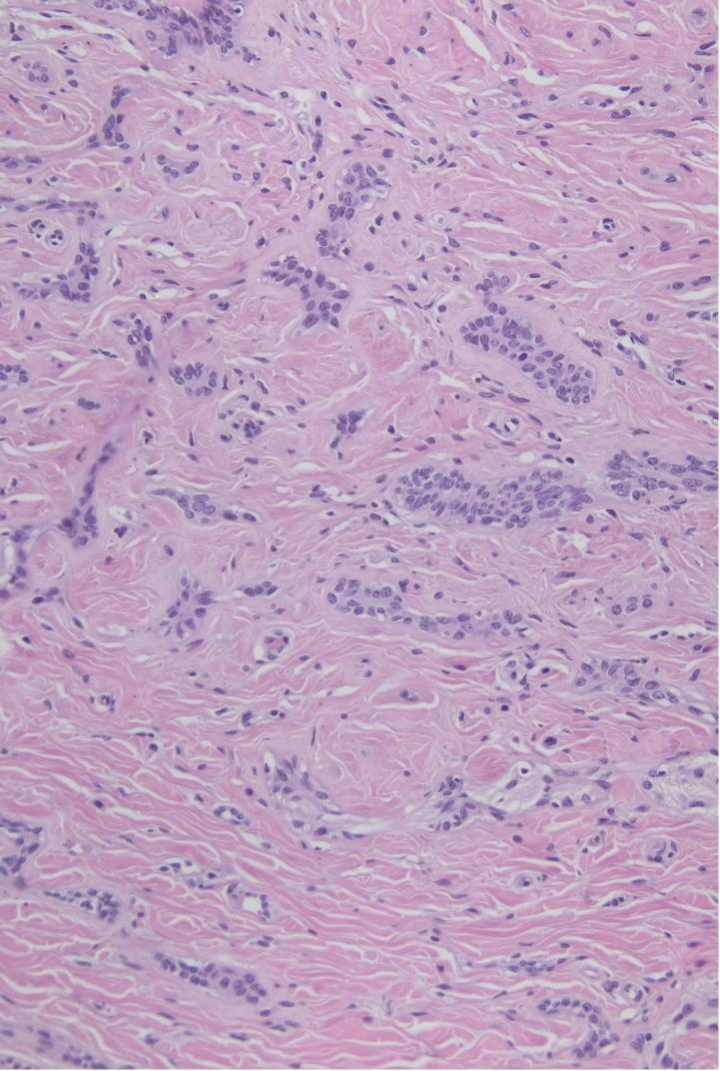
(e)

**Figure:**
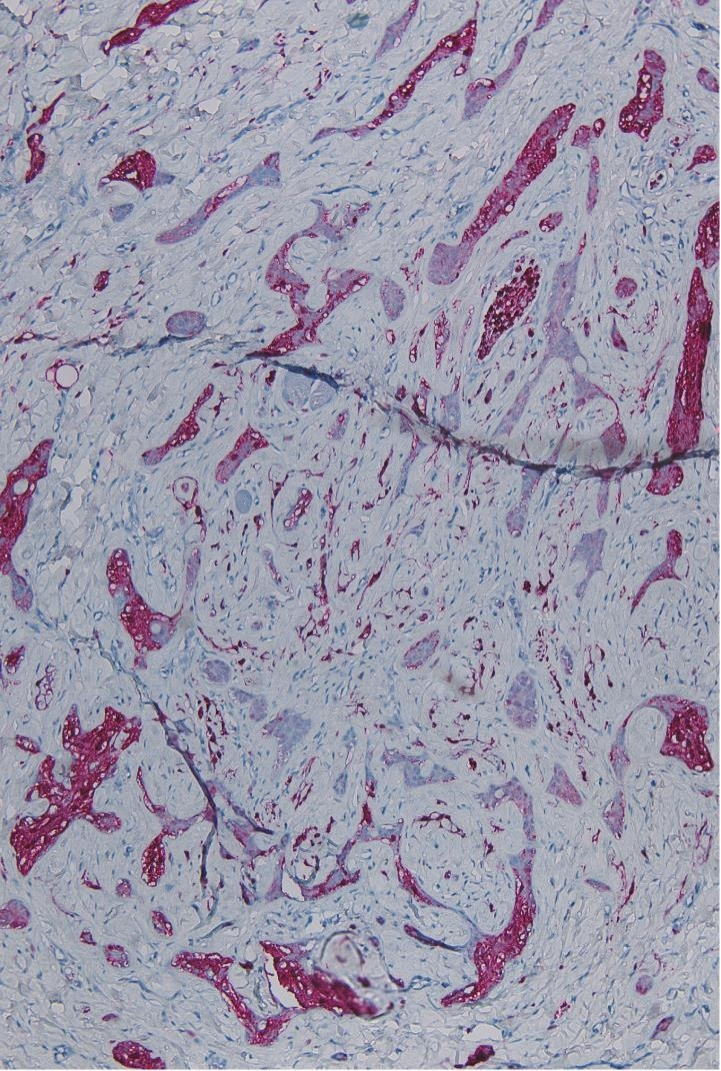
(f)

**Figure:**
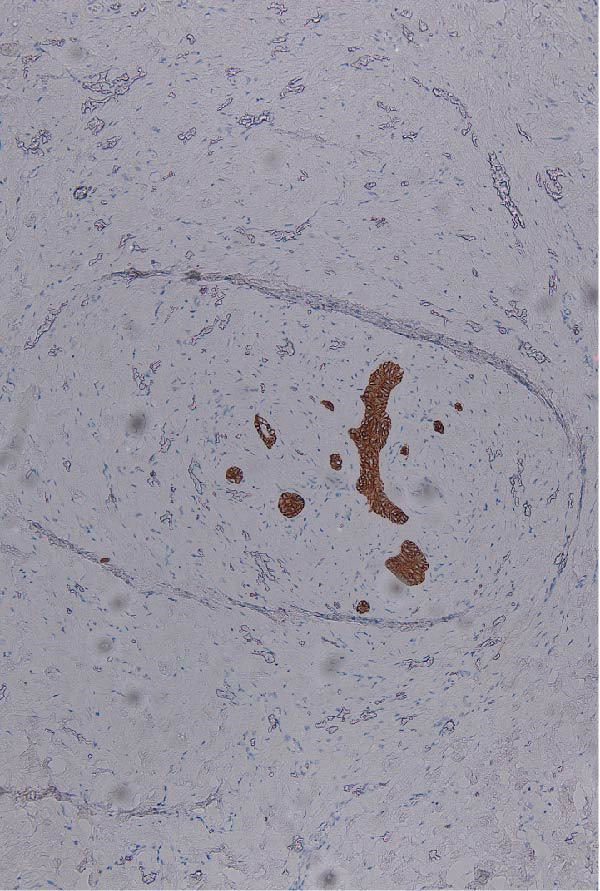
(g)

**Figure:**
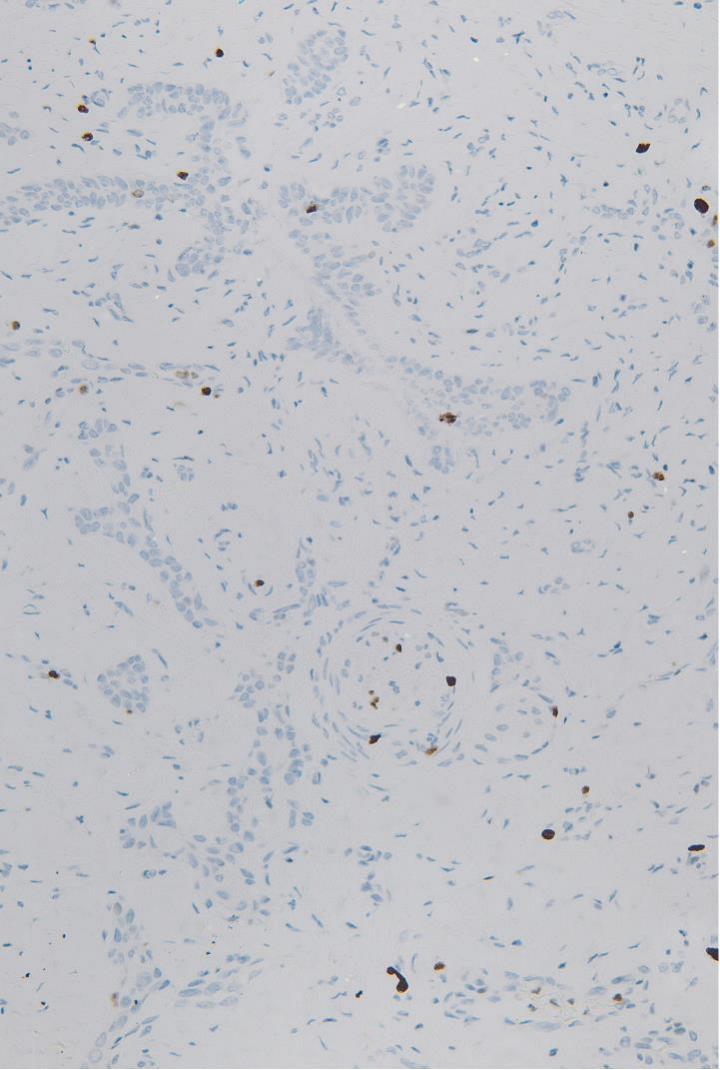
(h)

**Figure 4:**
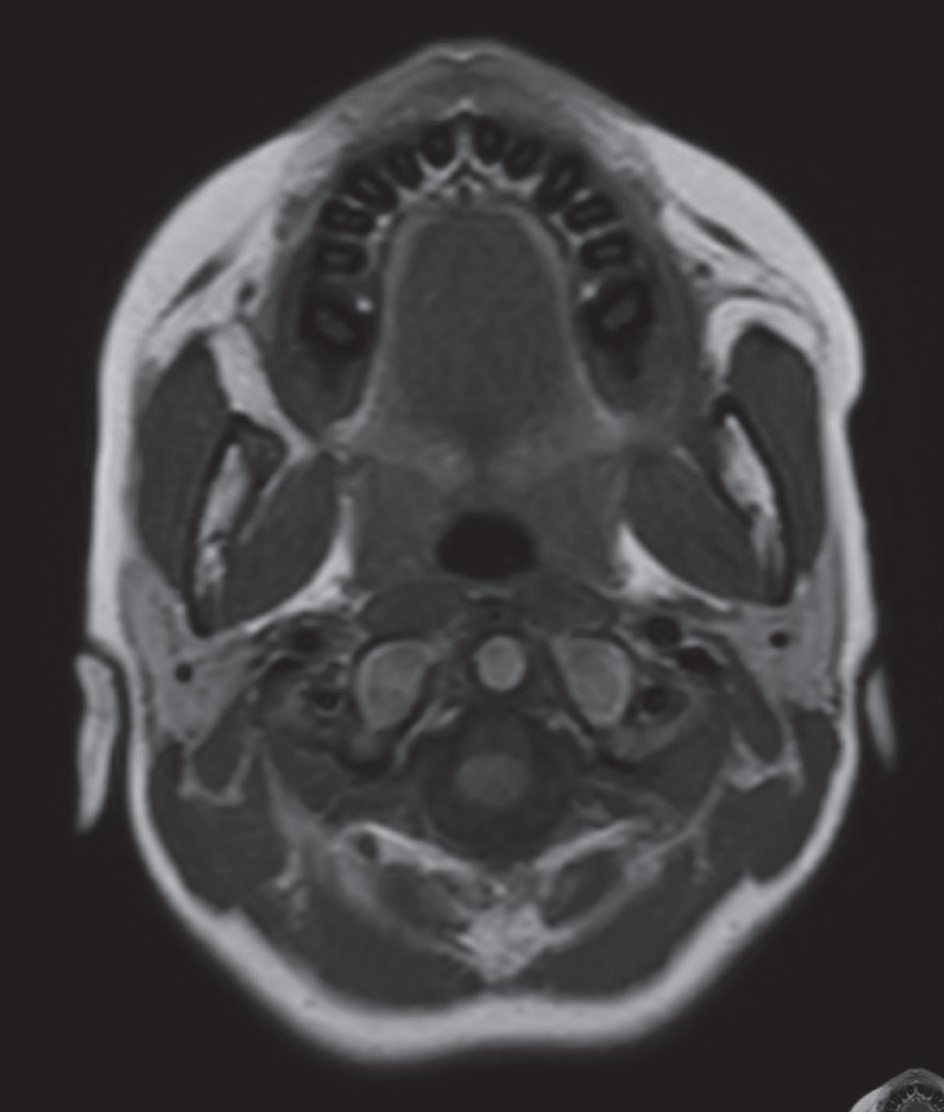
Postoperative MRI.

Given the successful surgical removal of the sialoblastoma with clear margins, we decided to proceed with clinical and radiological follow‐up of the patient and to abstain from radiotherapy or chemotherapy treatment. The radiological follow‐up protocol included MRI scans of the facial region every 4 months during the first postoperative year, every 6 months during the second year, and annually thereafter, for a total follow‐up period of 5 years, to rule out local recurrence or lymph node metastasis. This schedule was established considering the absence of anaplastic features and the completeness of tumor resection; otherwise, follow‐up would have been extended to 10 years, as recommended in the literature [8] (Figure 4). In addition, a chest X‐ray was performed once a year to exclude the development of pulmonary metastases. The patient complied with all scheduled follow‐up visits, and, to date, both imaging studies have yielded negative results. According to the patient’s parents, the surgical outcome was satisfactory, and the child has not experienced any functional or esthetic issues during the follow‐up period.

## 3. Discussion

Primary salivary gland tumors are rare in infancy and childhood, which is reported to account for only 2%–5% of all salivary gland neoplasms [9].

The diagnosis of sialoblastoma typically occurs during infancy, as 80% of cases are congenital tumors. During intrauterine life it may be detected through obstetric ultrasound. Occasionally, diagnosis may occur during the second decade of life [2]. No gender predilection has been reported [1].

The parotid gland is the most commonly affected site (45.2%), followed by the submandibular gland (29.0%) and minor salivary glands (25.8%) [2]. Therefore, the case of our 9‐year‐old patient with involvement of the minor salivary glands is relatively uncommon.

Clinically, sialoblastoma often presents as a palpable mass, occasionally associated with pain or, in severe cases, neurological symptoms such as facial paralysis or sensory impairments [2]. However, most cases, including ours, are asymptomatic. Metastases are infrequent, occurring in 19% of cases, with the lungs as the primary site, followed by laterocervical lymph nodes. Notably, metastases have been reported exclusively in parotid sialoblastoma [2, 7]. Our patient underwent a chest X‐ray which showed no pulmonary metastases and MRI scans of the neck which did not identify any laterocervical lymph node metastases.

Imaging plays a key role in diagnosis. On ultrasound, sialoblastoma appears as a hypoechoic mass, though this modality has limitations in visualizing deep structures and tumor‐nerve relationships. False negatives can occur, particularly with encapsulated lesions [10, 11]. Computed tomography (CT) and MRI are considered gold standards, along with fine needle aspiration biopsy for diagnostic confirmation and potentially sialography [12]. CT typically reveals hypodense masses compared to brain tissue and isodense regions relative to muscle. MRI, in contrast, shows low‐to‐intermediate signal intensity on T1‐weighted images and slightly hyperintense areas on T2‐weighted images, reflecting the tumor’s high nucleus‐to‐cytoplasm ratio [13, 14]. In our patient, the lesion demonstrated hypointensity on T1‐weighted MRI sequences, consistent with prior reports. The primary differential diagnoses include hemangiomas and teratomas. Hemangiomas, the most common salivary gland lesion in infancy, exhibit hypervascularization on Doppler ultrasound and strong, homogeneous signal enhancement on CT and MRI with contrast, unlike sialoblastoma, which shows weak, heterogeneous enhancement [15]. Teratomas typically present as solid‐cystic, multiloculated masses with calcifications [16]. Diagnosis of sialoblastoma typically involves histological examination of tissue samples stained with hematoxylin‐eosin [17]. Histologically, sialoblastoma exhibits cribriform or solid patterns, characterized by by nests of basaloid cells with peripheral palisading and maturation toward the center, alongside partially formed ductal and pseudoductal spaces separated by fibromyxoid stroma. Immunohistochemical analysis frequently detects cytokeratin, S‐100 protein, smooth muscle actin, and calponin [2, 18, 19]. Prognostic factors include the Ki‐67 proliferation index and p53 expression levels, where elevated values suggest a poorer prognosis [20]. In our case, the tumor exhibited a cribriform pattern with low malignancy features, including a low mitotic index and no necrosis, indicating a reduced risk of recurrence [21].

There is ongoing debate about whether sialoblastoma should be classified as benign or malignant. Many authors argue it should be viewed as a single entity with locally aggressive behavior that intensifies with growth. Tumor stage at diagnosis is considered the primary prognostic factor, underscoring the need for prompt intervention. Surgical resection stands as the primary approach and is curative when margins are clear [17]. Incomplete excision can lead to local invasion, recurrence, and, in severe cases, fatal outcomes [22]. When complete excision is unfeasible, chemotherapy and radiotherapy may be considered. However, radiotherapy’s use in pediatric patients is limited due to side effects such as mutagenesis and abnormal bone growth [23, 24]. Chemotherapy, reserved for extensive, metastatic, or resistant cases, has shown efficacy in treating pulmonary metastases. Despite the lack of standardized regimens, drugs such as vincristine, cyclophosphamide, doxorubicin, and carboplatin are commonly employed [9, 25]. In our case, the initial approach involved surgical excision of the lesion, achieving complete resection with clear margins. Consequently, additional therapies such as radiotherapy or chemotherapy were not deemed necessary for the patient at this time.

Recurrence or persistence of sialoblastoma occurs in approximately 25% of cases, typically within 3–17 months following the initial diagnosis. Despite this, the prognosis remains generally favorable, with a 5‐year overall survival (OS) rate of 95.5% and a disease‐free survival (DFS) rate of 68.1% [2]. To date, only four deaths directly attributed to sialoblastoma have been reported in the literature [9]. In our case, the patient will undergo regular follow‐up to enable the early detection of any potential recurrence.

The strength of this report lies in documenting an extremely rare tumor with detailed histopathological and immunohistochemical evaluation. Its main limitation is the nature of a single case study, and the relatively short follow‐up period, which prevents definitive conclusions on long‐term outcomes.

This case report has been prepared in accordance with the CARE (CAse REport) guidelines (Supporting Information).

## 4. Conclusions

The sialoblastoma is indeed considered a rare tumor of the salivary glands, and as such, its clinical management is controversial due to the lack of a significant number of cases reported in the medical literature. Without enough clinical data, it becomes difficult to establish a standardized treatment protocol that can be uniformly applied.

Based on clinical experiences and documented cases so far, radical surgical resection remains the first‐line treatment for sialoblastoma, with the possibility of avoiding additional treatments such as radiotherapy or chemotherapy and the associated side effects if radicalization is complete.

However, it is important to note that each case of sialoblastoma may be unique and require a personalized approach based on its specific clinical presentation and the characteristics of the patient.

## Author Contributions

Conceptualization: Giorgio Novelli, Gabriele Canzi, and Davide Sozzi. Methodology: Giorgio Novelli, Gabriele Canzi, and Camillo Di Bella. Writing – original draft preparation: Giulia Mangini and Chiara Picozzi. Writing – review and editing: Giulia Mangini, Chiara Picozzi, and Giorgio Novelli.

## Funding

No funding was received for this research. Open access publishing facilitated by Universita degli Studi di Milano‐Bicocca, as part of the Wiley ‐ CRUI‐CARE agreement.

## Disclosure

All authors have read and agreed to the published version of the manuscript.

## Ethics Statement

Ethical approval was not required for this case report, in accordance with the policies of our institution. Written informed consent for publication of clinical details and images was obtained from the patient’s parents.

## Conflicts of Interest

The authors declare no conflicts of interest.

## Supporting Information

Additional supporting information can be found online in the Supporting Information section.

## Supporting information


**Supporting Information** This case report has been prepared in accordance with the CARE (CAse REport) guidelines. The CARE checklist was used to ensure the completeness and transparency of reporting.

## Data Availability

All data are included in this manuscript.
