# Supplementary material for: Sialoblastoma of the Minor Salivary Glands: A Case Report
Source: Case Rep Dent. 2026 Jun 20;2026:5435950. doi: 10.1155/crid/5435950 (PMC13283031; doi:10.1155/crid/5435950)
Supplement: Supplementary file 1 — Supporting Information This case report has been prepared in accordance with the CARE (CAse REport) guidelines. The CARE checklist was used to ensure the completeness and transparency of reporting. [file CRID-2026-5435950-s001.pdf]

CARE
case report guidelines

CARE Checklist of information to include when writing a case report

(cc) BY-NC-ND

Topic	Item	Checklist item description	Reported on Line
Title	1	The diagnosis or intervention of primary focus followed by the words "case report"	2
Key Words	2	2 to 5 key words that identify diagnoses or interventions in this case report, including "case report"	16 - 17
Abstract (no references)	3a	Introduction: What is unique about this case and what does it add to the scientific literature?	19 - 20
	3b	Main symptoms and/or important clinical findings	23 - 24
	3c	The main diagnoses, therapeutic interventions, and outcomes	24 - 30
	3d	Conclusion—What is the main "take-away" lesson(s) from this case?	36 - 38
Introduction	4	One or two paragraphs summarizing why this case is unique (**may include references**)	42 - 50
Patient Information	5a	De-identified patient specific information.	53
	5b	Primary concerns and symptoms of the patient.	53 - 54
	5c	Medical, family, and psycho-social history including relevant genetic information	53 - 55
	5d	Relevant past interventions with outcomes	
Clinical Findings	6	Describe significant physical examination (PE) and important clinical findings.	56 - 58
Timeline	7	Historical and current information from this episode of care organized as a timeline	55 - 76
Diagnostic Assessment	8a	Diagnostic testing (such as PE, laboratory testing, imaging, surveys).	58 - 75
	8b	Diagnostic challenges (such as access to testing, financial, or cultural)	58 - 59
	8c	Diagnosis (including other diagnoses considered)	79 - 88
	8d	Prognosis (such as staging in oncology) where applicable	135 - 137
Therapeutic Intervention	9a	Types of therapeutic intervention (such as pharmacologic, surgical, preventive, self-care)	76 - 78
	9b	Administration of therapeutic intervention (such as dosage, strength, duration)	
	9c	Changes in therapeutic intervention (with rationale)	89 - 91
Follow-up and Outcomes	10a	Clinician and patient-assessed outcomes (if available)	94 - 96
	10b	Important follow-up diagnostic and other test results	91 - 94
	10c	Intervention adherence and tolerability (How was this assessed?)	93 - 94
	10d	Adverse and unanticipated events	94 - 96
Discussion	11a	A scientific discussion of the strengths AND limitations associated with this case report	159 - 162
	11b	Discussion of the relevant medical literature **with references**.	99 - 157
	11c	The scientific rationale for any conclusions (including assessment of possible causes)	169 - 171
	11d	The primary "take-away" lessons of this case report (without references) in a one paragraph conclusion	165 - 173
Patient Perspective	12	The patient should share their perspective in one to two paragraphs on the treatment(s) they received	94 - 96
Informed Consent	13	Did the patient give informed consent? Please provide if requested	Yes ☒ No ☐
